# Evidence for the influence of the milk fat globule membrane on bifidobacteria metabolism and cell surface properties

**DOI:** 10.3168/jdsc.2025-0873

**Published:** 2025-10-24

**Authors:** Celeste Miller, Rafael Jiménez-Flores

**Affiliations:** Department of Food Science and Technology, Parker Food Science and Technology, The Ohio State University, Columbus, OH 43210

## Abstract

*Bifidobacterium*, a genus commonly found in the microbiome of healthy infants, has been demonstrated to exert various beneficial effects on the gastrointestinal tract, contributing to overall health. Notably, these bacteria possess the ability to use complex glycoconjugates as nutrients, which holds importance for breast-fed infants, as human milk contains substantial quantities of these glycoconjugates. However, the underlying mechanisms of action of these beneficial properties remain largely unexplored. This study presents an alternative perspective on the nutrients in the diet that could potentially support the maintenance of a healthy *Bifidobacterium* population. We present evidence suggesting that the presence of milk fat globule membrane (MFGM), a component shared by all mammalian milk, significantly alters the metabolism of these bacteria. This alteration is reflected in the production of exopolysaccharides on the surface and secretome of the bacteria fed MFGM, whereas the absence of this component does not result in such changes. To substantiate this hypothesis, we employed proteomic and physicochemical methods, culminating in the evidence of the MFGM layer through electron microscopy.

## Highlights

• The MFGM affects the protein expression of *B. infantis*.
• *B. infantis* increases secretion of extracellular polysaccharides with digested milk.
• *B. infantis* cell surface charge is decreased with MPL.
• MPL interact with and thicken the surface morphology of *B. infantis*.
• *B. infantis* metabolic physiology changes in response to MFGM.

## Graphical Abstract

**Figure:**
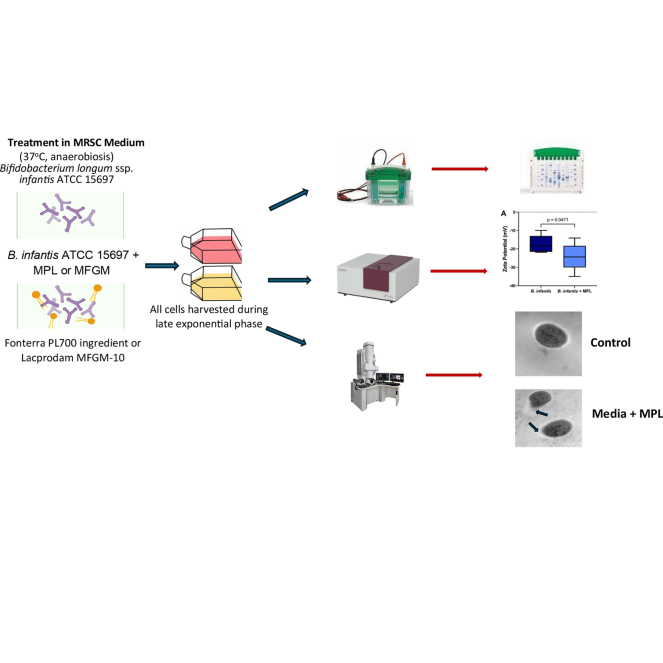
**Summary:***Bifidobacterium longum* ssp. *infantis* (*B. infantis*) is characteristic of a healthy breastfed infant microbiome that uses breast milk components to exert its beneficial effects on the host. *Bifidobacterium longum* ssp. *infantis* ATCC 15697 was grown in de Man, Rogosa, and Sharpe medium with 0.05% cysteine (MRSC) supplemented with either milk phospholipids (MPL) or digested infant formula containing milk fat globule membrane (MFGM). Samples of bacterial pellets and supernatants were collected during the exponential phase of growth when metabolism is highest. The bacterial cell metabolism was analyzed with proteomics, along with microscope imaging and cell surface charge evaluation. The bacterial supernatant was analyzed for exopolysaccharide production. Metabolism of *B. infantis* was altered in the presence of MFGM, where the protein expression was influenced, cell surface morphology was thicker and accompanied by a decrease in surface charge, and exopolysaccharide production increased. These results indicate MPL components may enhance the metabolism of *B. infantis* for increased host benefits.

*Bifidobacterium* is an anaerobic, gram-positive, nonmotile group of bacteria that is associated with the gut microbiome of breast-fed infants. These bacteria possess the ability to use human milk glycoconjugates. Bifidobacteria are substantially more abundant in the infant microbiota than in adults, and this suggests they have an important role for early life development (Stuivenberg et al., 2022). The dominance of bifidobacteria results in competitive exclusion of harmful bacteria; bifidobacterial cell surfaces contain adhesion proteins that encourage their interaction with epithelial cells, which then limits space and nutrient availability for pathogens (Stuivenberg et al., 2022).

In utilizing glycoconjugates, bifidobacteria produce metabolites that are beneficial for the host and for the microbiota composition. Short-chain fatty acids (**SCFA**) are waste products from microbial fermentation of indigestible glycoconjugates; therefore, glycoconjugates are an important fuel source for commensal bacteria. *Bifidobacteria* produce SCFA including acetate and lactate, which serve to reduce the lumen pH for the prevention of pathogen growth, and butyrate, which is the main energy source for colonocyte development. Colonocytes are specialized cells that serve as an important factor to maintain the intestinal barrier integrity and absorb nutrients (Stuivenberg et al., 2022). Utilization of human milk glycoconjugates also results in the production of intermediate fermentation products that may be used by other commensal bacteria to establish cross-feeding networks. Ultimately, bifidobacteria are important for proper sequential establishment of the infant gut microbiome composition. There is also correlation between bifidobacterial colonization and a reduction in allergy and atopic diseases in infants, indicating that bifidobacteria may play a role in encouraging immune system maturation (Stuivenberg et al., 2022). Overall, bifidobacteria are important for the development of both the gastrointestinal tract (**GIT**) and immune system in infants. However, to exert their effects, these bacteria must first be able to survive and colonize in the GIT.

Bifidobacteria possess a unique fermentation pathway known as the “bifid shunt,” which includes enzymes for transport and metabolization of complex glycoconjugates present in human milk (Stuivenberg et al., 2022). These glycoconjugates are used as growth factors by bifidobacteria that serve to enhance the bacterial colonization and adherence in the GIT (Stuivenberg et al., 2022). Particularly, glycoconjugates in human milk are associated with the milk fat globule membrane (**MFGM**), which, in nonhomogenized milk, is a bioactive tri-phospholipid layer membrane that serves to emulsify fat globules and deliver lipid soluble nutrients. It contains complex proteins, essential lipids, and a multitude of glycoconjugates known to have an important role in infant development including enrichment of the gut microbiome (Brink and Lönnerdal, 2020). A study conducted by Kim et al. (2025) demonstrated the growth of *Bifidobacterium bifidum* on the retentate of whey protein phospholipid concentrate containing an abundance of milk glycoconjugates as the sole carbon source for the bacteria. An abundance of SCFA was produced along with an increase in the expression of multiple glycosyl hydrolases, which indicates the use of these nutrients by bifidobacteria (Kim et al., 2025). The medium used in this study was produced in a similar manner to the isolation of MFGM from milk. These findings support the idea of MFGM-associated glycoconjugate use by bifidobacteria to produce intermediate compounds for cross-feeding network establishment and metabolite production beneficial for the host.

Furthermore, in vitro cultures of bifidobacteria with MFGM components have demonstrated significantly greater bacterial cell density along with the increase in glycosyl hydrolase expression for enhancement of energy and carbohydrate metabolism (Zhao et al., 2022). The adhesive factors present on bifidobacterial cell surfaces also encourage their interaction with MFGM components (Yadav et al., 2022). Although these studies demonstrate the selective nature of milk glycoconjugate use by these bacteria in vitro, they do not reveal how bifidobacteria may be able to survive through the GIT environment to colonize the infant gut. Studies have compared breast-fed infant stool with that of formula-fed infants and formula with the addition of MFGM, which demonstrate a microbiota similar to that of breast-fed infants with the addition of MFGM (Le Huërou-Luron et al., 2018; Yadav et al., 2022; Zhao et al., 2022). The supplementation of MFGM to infant formulas (**IF**) not only reveals a greater relative abundance of bifidobacteria but also a negative correlation with *Escherichia* in infant stool samples indicating a selective preference for a beneficial microbiome (Zhao et al., 2022). Therefore, there is a mechanism by which MFGM may be enhancing bacterial survival through the harsh digestive tract.

Additionally, it has been demonstrated that the combination of MFGM and bifidobacteria improves the bacterial cell viability, integrity, and metabolic function when exposed to bile salts and in vitro digestion (Kosmerl et al., 2023; Zhang et al., 2024). Zhang et al. (2024) demonstrated that MFGM addition to *Bifidobacteria longum* ssp. *infantis* culture increased bacterial survival when exposed to bile salts. Specifically, there was an overexpression of bile salt efflux transporters and a restoration of nutrient transport and energy metabolism genes compared with culture without MFGM (Zhang et al., 2024). Furthermore, Kosmerl et al. (2023) demonstrated a significant increase in *B. infantis* survival under simulated digestion conditions with the addition of milk phospholipids compared with the control. Once again, this indicates MFGM enhances the protection of bifidobacteria.

One potential protective mechanism of *B. infantis* is its ability to produce polysaccharides that can either stay attached to the bacterial cell or be released into the medium. The attached polysaccharides may serve to protect the cell through harsh environments including the digestive tract, whereas the released exopolysaccharides (**EPS**) may serve to benefit the host or establish cross-feeding networks for the microbiome (Kosmerl et al., 2023). It is known that the MFGM improves *Bifidobacterium* viability when exposed to bile salts and that a physical interaction with MFGM may facilitate bacterial adhesion to the intestinal mucosal layer for colonization and growth (Kosmerl et al., 2023; Zhang et al., 2024). However, there is a gap in understanding what the direct impacts of MFGM on *B. infantis* physiology may be, including how it aids in bacterial cell protection, colonization, and growth in the GIT as part of a healthy breast-fed infant microbiome. Therefore, the objectives of this work were to first characterize how digested MFGM influenced *B. infantis* protein expression and correlate that to the production of EPS, and second to determine the effect of milk phospholipids on *B. infantis* bacterial cell surface properties. We hypothesized that the MFGM initiates a metabolic change in *B. infantis* behavior to enhance its survival and growth for the microbiome.

Two commercial IF, one milk based (**MIF**) and one soy based (**SIF**), were prepared according to container instructions with 0%, 2%, or 10% wt/wt supplementation with a commercial whey-derived MFGM ingredient Lacprodan MFGM-10 (Arla Foods Ingredients, Copenhagen, Denmark). The formulas underwent static in vitro digestion following a modified INFOGEST 2.0 digestion guidelines tailored for infant digestion simulation (Ménard et al., 2018). *Bifidobacterium* longum ssp. *infantis* ATCC 15697 was purchased from the American Type Culture Collection (ATCC, Manassas, VA) and inoculated from glycerol stocks into de Man, Rogosa, Sharpe broth with 0.05% l-cysteine (**MRSC**) and covered with sterile mineral oil for anaerobic conditions at 37°C. The strain was then subcultured twice with 0%, 0.5%, 1%, or 2% of IF digesta including a digestive enzyme (**DE**) control for experiments. For protein analysis according to Abdelhamid and Yousef (2024), *B. infantis* bacterial pellets were washed twice with PBS and resuspended in a 1:1 methanol and water solution. The resuspended pellet was then added to a screw-cap tube with sterile glass beads (150 mg of 0.1-mm beads and 150 mg of 0.5-mm beads) and homogenized for 9 min in a bead mill using 1.5-min intervals at 5 m/s to lyse the cells and release the protein. The mixture was then centrifuged and supernatant collected for protein analysis using SDS-PAGE on a 12% hand-cast polyacrylamide gel. Coomassie blue staining was performed followed by destaining with 10% acetic acid for visualization of protein bands. Protein bands were then cut and sent for sequencing at the Campus Chemical Instrumentation Center Mass Spectrometry and Proteomics Facility at The Ohio State University. Briefly, the bands underwent protease digestion before identification using liquid chromatography-MS/MS where the data files were searched using Mascot Daemon by Matrix Science version 2.2.1 (Boston, MA) on a 16-node IBM blade system. The proteins identified were then checked manually to ensure a Mascot score of 50 or higher with a minimum of 2 unique peptides from one protein having a -b or -y ion sequence tag of 5 residues or better are accepted.

The growth curve of *B. infantis* was measured for all medium conditions following subcultures with IF digesta and DE at 600 nm on a microplate reader (Multiskan GO, Thermo Fisher, Waltham, MA) for 24 h against medium blanks after adjusting the pellet optical density at 600 nm (**OD_600_**) to 0.1 ± 0.01. Fresh cultures were then prepared for sample collection at 3, 5, 9, and 24 h after inoculation corresponding to the initial, growth, early stationary, and late stationary phases of growth for EPS analysis. The cell-free supernatant of each sample was collected and filtered using 0.22-μm syringe filter. To determine the concentration of total sugars, the phenol sulfuric acid assay was performed. Briefly, filtered supernatant was diluted 1:500 in deionized water. One milliliter of sample was mixed with 0.6 mL of 5% phenol by vortex for 30 s; next, 3.6 mL of concentrated sulfuric acid was added to the mixture and vortexed for 30 s, then measured at 480 nm on a microplate reader. For reducing sugar determination, dinitro salicylic acid (**DNSA**) assay was used. A solution of 2 *M* sodium hydroxide and 300 g of sodium potassium tartrate were warmed to 50°C with constant mixing, then 10 g of 3,5-dinitro salicylic acid was added to the warmed mixture with continuous stirring; the solution was cooled to room temperature and filtered through filter paper into an amber bottle. Water was added to bring the mixture to 1 L, and the solution was stored at room temperature protected from light. Filtered supernatant was diluted 1:100 in deionized water and 3 mL of sample was combined with 1 mL of DNSA solution. The samples were placed in a boiling water bath for 5 min, then measured at 540 nm on a microplate reader. For both sugar assays, the sugar concentration was determined by using a standard curve of glucose to galactose 1:1 ratio. To determine EPS, the result of the DNSA assay was subtracted from the phenol sulfuric acid assay to give the concentration of nonreducing sugars.

To measure the zeta potential according to Kosmerl et al. (2023), *B. infantis* was subcultured twice in MRSC with 0.5% of a milk phospholipid (**MPL**) ingredient originally isolated from MFGM β-serum powder generously donated from Fonterra Co-operative Group Ltd. (PL700 Concentrate, Auckland, New Zealand). The bacterial pellet was adjusted to OD_600_ 0.1 ± 0.01 and measurements were recorded using a NanoBrook 90 Plus instrument (Brookhaven Instruments, Holtsville, NY) collected at 25°C using the Smoluchowski approximation. The refractive indices (**RI**) for gram-positive *Bacillus thuringiensis* bacterial cells (1.528) and RI for water (1.33) were used (Kosmerl et al., 2023).

Finally, for imaging, a concentrated suspension was prepared in PBS. This suspension was then mixed with a sterile, melted, low-gelling temperature agarose solution (4% at 60°C). After solidification, small squares (1–2 mm^2^) were cut using a scalpel. These squares were then fixed with 3% formaldehyde and incubated for 24 h. After carefully removing the supernatant, the samples were washed 5 times with PBS under gentle agitation (5 min each). The samples were then sent to the Campus Microscopy and Imaging Facility of The Ohio State University for transmission electron microscopy analysis. Each sample was fixed with 2.5% glutaraldehyde for 2 h, postfixed with 1% osmium tetroxide in 0.1 *M* phosphate buffer for 2 h, and then stained with 1% uranyl acetate for 1 h at room temperature. After dehydration in a graded ethanol series (50%, 70%, 80%, 90%, 100%), the samples were embedded in Eponate 12 resin. The samples were sectioned at 70 nm using an ultramicrotome (EM UC7, Leica Microsystems, Vienna, Austria) and stained with 2% uranyl acetate and Reynold's lead citrate. Finally, the samples were visualized using a FEI Tecnai G2 Spirit microscope (FEI Company, Hillsboro, OR) operated at 80 kV (Kosmerl et al., 2023).

*Bifidobacteria longum* ssp. *infantis* experiments were executed with duplicate cultures and 3 readings were taken per sample. Results are expressed as the mean ± SE. Significance for EPS was determined using a multiple comparisons 2-way ANOVA with Tukey Kramer post hoc pairwise comparisons test using R statistical software (version 4.3.2; R Core Team, 2023). Statistical analysis for zeta potential was conducted using a Student's *t*-test performed in GraphPad Prism V9.4 (GraphPad Software Inc.). A 95% CI was used and a *P*-value of <0.05 was considered significant.

In this work, we found that the presence of MFGM changes the protein expression in the bacteria, very likely due to change in metabolism. The growth of *B. infantis* with digested IF containing MFGM altered the bacterial physiology as evidenced by the changes in protein expression (Figure 1). We observed an alteration in chaperone protein expression when grown in the presence of digested MIF supplemented with 10% MFGM that was not demonstrated in digested SIF or the DE control. Chaperone proteins are always expressed by metabolically active cells as part of the fundamental physiology for bacterial survival under normal conditions as well as during stress (Alam et al., 2021). The 3 identified chaperone proteins—ClpB, DnaK, and GroEL—are all classified as heat shock proteins, which stabilize and assist protein refolding under stressful conditions. ClpB is an ATPase that works with DnaK to disaggregate stress-denatured proteins for survival of the bacterial cell in conditions like heat, low pH, starvation, and osmotic and oxidative stress (Alam et al., 2021). *Bifidobacteria longum* ssp. *infantis* grown with the addition of digesta containing MFGM decreased the expression of these chaperone proteins as GroEL and DnaK were not identified.

**Figure 1:**
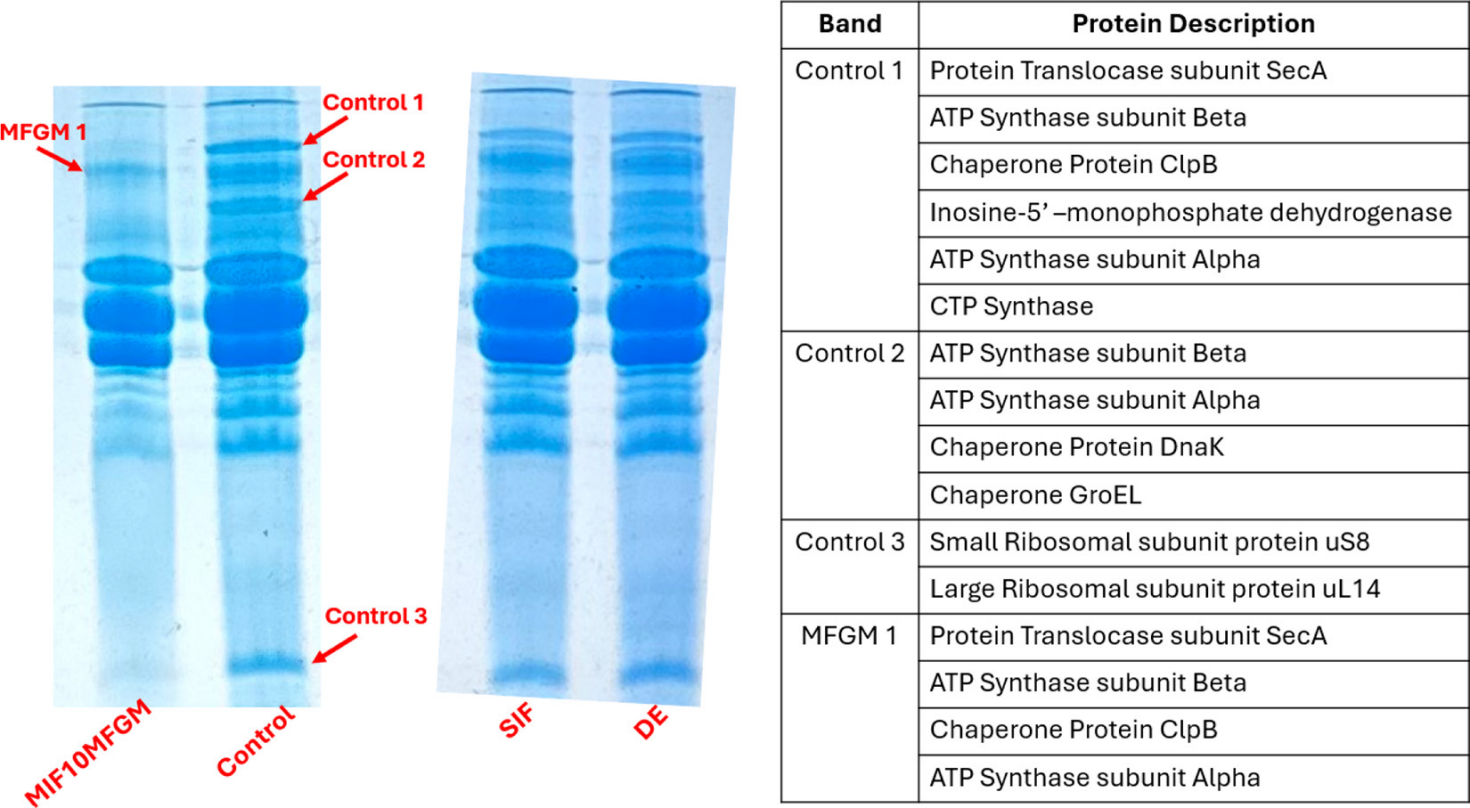
Sodium dodecyl sulfate-PAGE for *Bifidobacteria longum* ssp. *infantis* culture grown in MRSC (control), digested milk-based infant formula supplemented with 10% MFGM (MIF10MFGM), digested soy-based infant formula (SIF), and digestive enzyme control (DE). Four bands were cut and sent for sequencing indicated by the red arrows. Protein sequencing results are shown in the table on the right.

Another prominent change in protein expression was observed regarding the ribosomal proteins where the corresponding control 3 band was hardly visualized, presumably by a downregulation, in the sample of *B. infantis* grown with IF containing MFGM. The ribosome is the key machinery of the cell responsible for protein synthesis where both the large and small subunits must come together for mRNA translation into a protein. The large ribosomal subunit uL14 is largely conserved between all kingdoms of life and is the primary binding site of initiation factor 6, which is part of the mechanism responsible for bringing the large and small ribosomal subunits together for protein synthesis (Khusainov et al., 2020; Yeh et al., 2024). This finding suggests that *B. infantis* grown with digesta containing MFGM was also downregulating its protein synthesis mechanisms. *Bifidobacteria longum* ssp. *infantis* demonstrated a change in physiology in response to the additional nutrients provided in the medium. Because the digesta does contain protein, this change in physiology may have reduced the need for the bacteria to produce proteins itself, and therefore a reduction in ribosomal proteins was observed. Both samples expressed fundamental metabolic enzymes involved in energy and regulation as the type 2 secretion protein subunit SecA and ATP synthase were observed (Papanikou et al., 2007). This finding indicates that the bacterial cells were metabolically active under both control and digesta supplementation conditions. However, we observed a decrease in chaperone protein and ribosomal protein expression, which suggests that the bacteria are expending greater metabolic energy elsewhere as the bacteria grown with digesta containing MFGM still expressed proteins required for energy output.

This work also provides data that the presence of MFGM in media increases the production of EPS in the supernatant of the bifidobacteria culture. Because the cultures were grown with digested nutrients containing glycoconjugates and DE, we expected there to be an increase in polysaccharide production for bacterial protection to ready the cell for attachment to the intestinal epithelia and for cross-feeding network establishment. The change in protein expression in response to the addition of MIF digesta containing MFGM correlates with a change in bacterial metabolite production. Figure 2 displays EPS production by *B. infantis* with various IF digesta supplementations. The EPS concentrations in the bacterial secretome can be correlated with the change in basal protein expression where the control, SIF, and DE samples had similar EPS production (Figure 2) as well as protein profiles that were unchanged in the SDS-PAGE (Figure 1). Significantly (*P* = 0.01102) greater EPS production was demonstrated with *B. infantis* grown with MIF containing 10% MFGM supplementation, and this result correlates with a change in the protein profile. This greater EPS production may suggest that *B. infantis* is expending more metabolic energy for producing EPS rather than toward the expression of chaperone and ribosomal proteins. *Bifidobacteria longum* ssp. *infantis* can use milk glycoconjugates via the bifid shunt and the required enzymes for this activity are upregulated in the presence of glycoconjugate substrates (Zhao et al., 2022; Kim et al., 2025). Our findings align with this understanding, as *B. infantis* demonstrated the greatest metabolite production with the addition of digested MIF with MFGM where MFGM is bringing in additional glycoconjugates for upregulation of bifid shunt enzymes.

**Figure 2:**
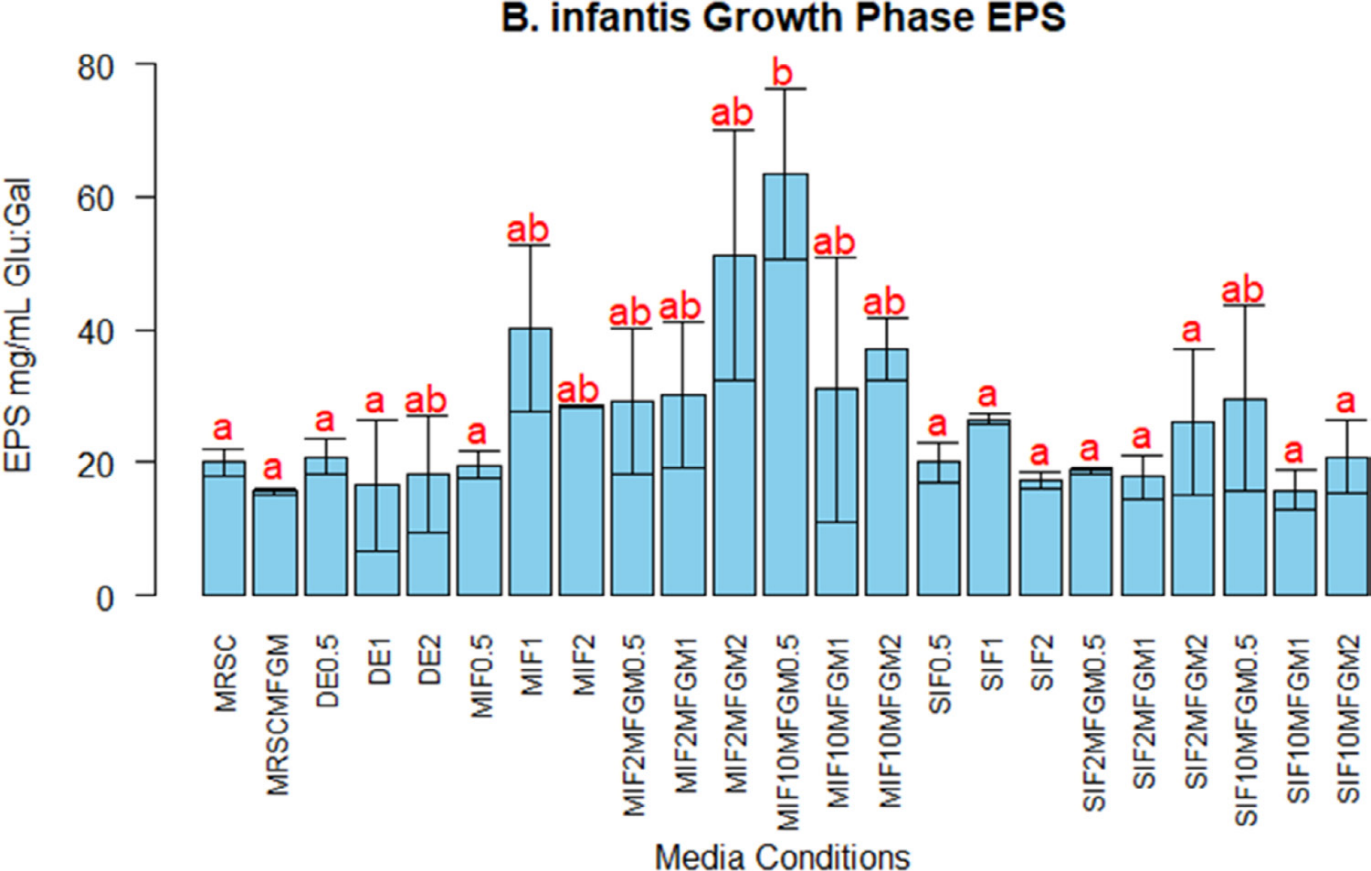
The production of exopolysaccharides (EPS) of *Bifidobacteria longum* ssp. *infantis* under different growth medium supplementation conditions: MRSC; MRSC + 0.5% MFGM (MRSCMFGM); digestive enzymes added at 0.5%, 1%, and 2% (DE0.5, DE1, DE2); digested milk-based infant formula (MIF) added at 0.5%, 1%, and 2% (MIF0.5, MIF1, MIF2); digested MIF with 2% MFGM added at 0.5%, 1%, and 2% (MIF2MFGM0.5, MIF2MFGM1, MIF2MFGM2); digested MIF with 10% MFGM added at 0.5%, 1%, and 2% (MIF10MFGM0.5, MIF10MFGM1, MIF10MFGM2); digested soy-based infant formula (SIF) added at 0.5%, 1%, and 2% (SIF0.5, SIF1, SIF2); digested SIF with 2% MFGM added at 0.5%, 1%, and 2% (SIF2MFGM0.5, SIF2MFGM1, SIF2MFGM2); and digested SIF with 10% MFGM added at 0.5%, 1%, and 2% (SIF10MFGM0.5, SIF10MFGM1, SIF10MFGM2). Significance is indicated by different letters where *P* < 0.05. Values shown are average ± SD.

The production of EPS also corresponded to a physical change in the bacterial cell surface characteristics including a decrease in the zeta potential and a thickening of the outer cell surface layer as seen in Figure 3. Gram-positive bacterial cell surface charge is accredited to surface proteins and carbohydrates that can be altered due to environment or nutritional changes (Kosmerl et al., 2023). The significant (*P* = 0.0471) decrease in zeta-potential or surface charge in response to *B. infantis* grown with MPL indicates the potential attachment of these phospholipids to the bacterial cell. Bifidobacteria have a negative surface charge due to their gram-positive phenotype along with negatively charged polysaccharides present on the cell surface. This decrease in surface charge also correlates with the increase in thickness of the bacterial cell surface when grown with MPL (Figure 3), where this change in surface morphology resembles an increase in surface polysaccharides. Upon further analysis (data not shown), the bacterial outer layer did have changes in the lipid profile and carbohydrate polymers with MPL suggesting direct interaction with the phospholipids. Bacterial surface charge has been used as a predicter for adhesion to the intestinal mucosa and is correlated with lipid-mediated nutrient uptake (Halder et al., 2015; Ortega-Anaya et al., 2021). Alteration of bacterial surface characteristics is an important factor for microbiota establishment where interaction of the bacteria with the host mucosa is necessary for colonization, and this interaction is facilitated through cell surface proteins and polysaccharides.

**Figure 3:**
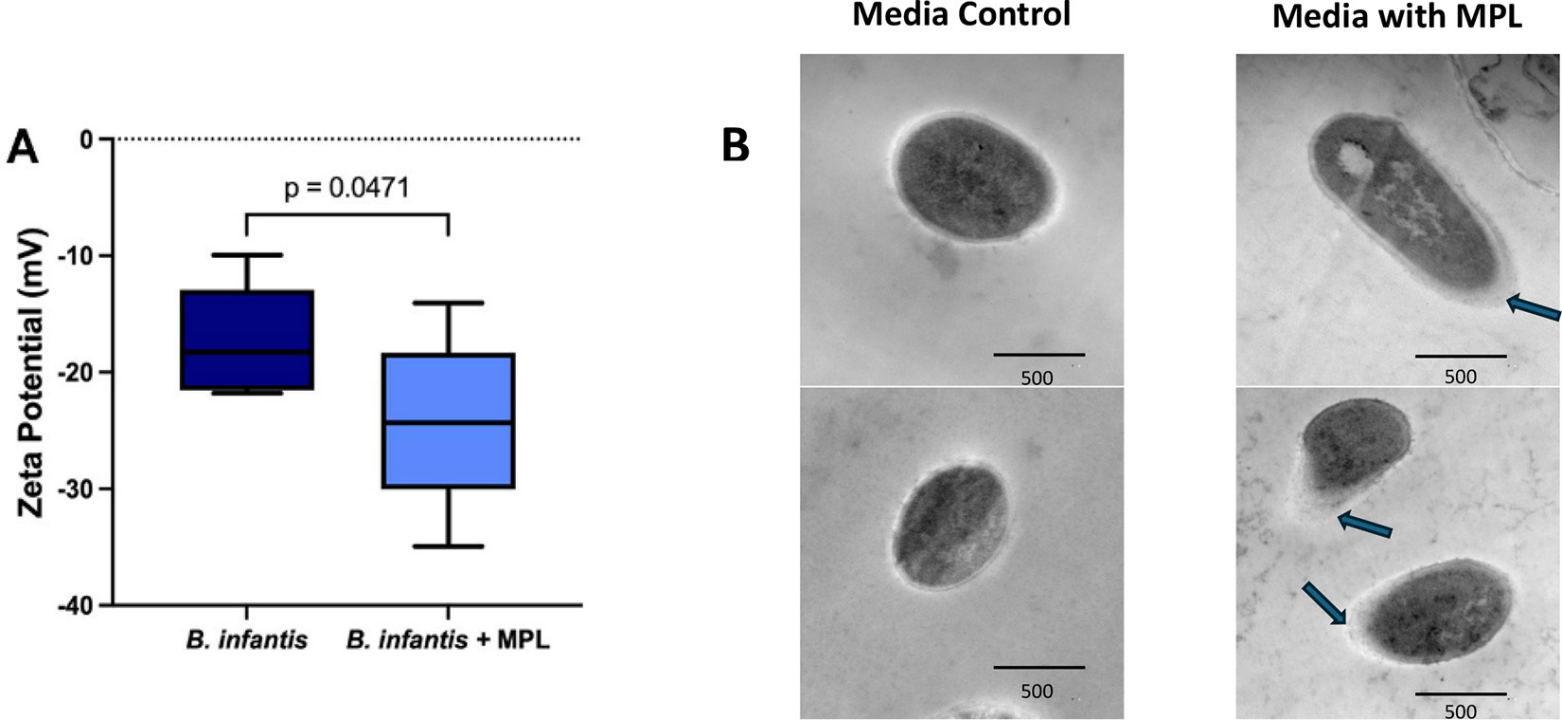
(A) Graph of the zeta-potential of the surface of *Bifidobacteria longum* ssp. *infantis* in the presence or absence of milk phospholipids (MPL) in the growth media. Vertical lines depict SD and the horizontal line is the median, with the rectangles indicating the main range of measurements. (B) Transmission electron micrographs of samples of *B. infantis*. Scale bars are 500 nm. The medium control images on the left show bacterial cells grown in MRSC medium only, and the images on the right depict *B. infantis* grown in MRSC with 0.5% MPL. The thickening of the outer cell surface due to the layer of exopolysaccharide produced in the presence of MPL ingredient is indicated with arrows.

From this work we can summarize that the MFGM may directly interact with the *B. infantis* bacterial cell to initiate enhancement of the bifid shunt pathway for MFGM nutrient use. The use of MFGM glycoconjugates may then subsequently alter *B. infantis* physiology for increased polysaccharide production to protect the bacterial cell through the digestive tract to survive, colonize, and establish cross-feeding networks for healthy infant microbiota establishment.
